# Diverse Viruses Carrying Genes for Microbial Extremotolerance in the Atacama Desert Hyperarid Soil

**DOI:** 10.1128/mSystems.00385-21

**Published:** 2021-05-18

**Authors:** Yunha Hwang, Janina Rahlff, Dirk Schulze-Makuch, Michael Schloter, Alexander J. Probst

**Affiliations:** a Department of Organismic and Evolutionary Biology, Harvard University, Cambridge, Massachusetts, USA; b Group for Aquatic Microbial Ecology, Environmental Microbiology and Biotechnology, Department of Chemistry, University of Duisburg-Essen, Essen, Germany; c Center for Astronomy and Astrophysics, Technische Universität Berlin, Berlin, Germany; d German Research Centre for Geosciences, Section Geomicrobiology, Potsdam, Germany; e Department of Experimental Limnology, Leibniz-Institute of Freshwater Ecology and Inland Fisheries, Stechlin, Germany; f Research Unit for Comparative Microbiome Analysis, Helmholtz Zentrum München, German Research Center for Environmental Health, Oberschleißheim, Germany; University of California, Davis

**Keywords:** auxiliary metabolic genes, viromics, metagenomics, viral dispersal, extremophiles, virus-host interactions

## Abstract

Viruses play an essential role in shaping microbial community structures and serve as reservoirs for genetic diversity in many ecosystems. In hyperarid desert environments, where life itself becomes scarce and loses diversity, the interactions between viruses and host populations have remained elusive. Here, we resolved host-virus interactions in the soil metagenomes of the Atacama Desert hyperarid core, one of the harshest terrestrial environments on Earth. We show evidence of diverse viruses infecting a wide range of hosts found in sites up to 205 km apart. Viral genomes carried putative extremotolerance features (i.e., spore formation proteins) and auxiliary metabolic genes, indicating that viruses could mediate the spread of microbial resilience against environmental stress across the desert. We propose a mutualistic model of host-virus interactions in the hyperarid core where viruses seek protection in microbial cells as lysogens or pseudolysogens, while viral extremotolerance genes aid survival of their hosts. Our results suggest that the host-virus interactions in the Atacama Desert soils are dynamic and complex, shaping uniquely adapted microbiomes in this highly selective and hostile environment.

**IMPORTANCE** Deserts are one of the largest and rapidly expanding terrestrial ecosystems characterized by low biodiversity and biomass. The hyperarid core of the Atacama Desert, previously thought to be devoid of life, is one of the harshest environments, supporting only scant biomass of highly adapted microbes. While there is growing evidence that viruses play essential roles in shaping the diversity and structure of nearly every ecosystem, very little is known about the role of viruses in desert soils, especially where viral contact with viable hosts is significantly reduced. Our results demonstrate that diverse viruses are widely dispersed across the desert, potentially spreading key stress resilience and metabolic genes to ensure host survival. The desertification accelerated by climate change expands both the ecosystem cover and the ecological significance of the desert virome. This study sheds light on the complex virus-host interplay that shapes the unique microbiome in desert soils.

## INTRODUCTION

Viruses are considered the most abundant biological entities on Earth (1), with high genomic diversity (2) and an expanding ecological and biogeochemical importance. Viruses, particularly bacteriophages, shape microbial community turnover and composition (3, 4), nutrient cycling (5, 6), and microbial evolution (7, 8) in marine (9) and freshwater (10) environments. Progress in soil virome studies is lagging compared to those in marine and gut microbiome systems (11–13), mainly due to the difficulties of isolating viruses from heterogeneous and complex soil environments (14). However, recent metagenomic approaches revealed diverse soil viruses in high abundance (15) which play significant roles in carbon processing (16–18) and other types of nutrient turnover (19, 20). Even less explored are viruses in extreme soil environments, where life itself becomes scarce in biomass and low in biodiversity (21–23). Understanding the abundance and diversity of viruses as well as their interactions with extremotolerant microbes in environments can highlight the unique roles viruses may play in driving the adaptation of their hosts and reveal the dispersal and diversification of viruses in sparsely populated and harsh environments.

Hyperarid desert soils are unique terrestrial environments, where low water availability limits proliferation and diversification of life. The biota that permanently inhabits these environments is often limited to a few bacterial and archaeal phyla. Recent studies of warm (i.e., the Namib Desert and Sahara Desert) and cold (i.e., Antarctic soil) hyperarid desert viromes have revealed abundant viruses of diverse lineages and sizes, with lysogenic and pseudolysogenic viruses being more prevalent than lytic viruses in warm deserts (21, 24–26). With little water availability and extended periods of drought, hyperarid desert soils present a distinct model for studying viral persistence and dispersal. In these ecosystems, viral mobility is limited compared to that in aquatic environments, in which both viruses and hosts freely diffuse (14).

The Atacama Desert is one of the harshest environments on Earth, with its hyperarid core experiencing extreme desiccation with a mean annual precipitation of <2 mm (27). The surface soil of the Atacama hyperarid core generally contains <1% (by weight) water and experiences high daily UV radiation (30 J⋅m^−2^) (28), extreme diurnal temperature fluctuations (∼60°C) (29), and additional osmotic pressure from the accumulation of salts (28, 30). Scarce populations of highly adapted microbial communities consisting of *Actinobacteria*, *Firmicutes*, *Chloroflexi* (28, 30, 31), and, more recently discovered, *Thaumarchaeota* (29) were found to inhabit soils of the Atacama hyperarid core. However, very little is known about viruses from these desert soil microbiomes. Crits-Christoph et al. (32) identified viral sequences and their potential hosts in halite endoliths of the Atacama. Additionally, Uritskiy et al. (33) detected transcriptionally active viruses potentially infecting *Halobacteria* also inhabiting halite salt nodules in a salar located in the Atacama Desert. These niche halite host-virus relationships highlight the need to characterize the impact of viruses in broad desert soils that represent one of the largest and rapidly expanding terrestrial ecosystems on the planet (∼35% of the Earth’s land surface [34]). In our previous study (28), we also detected viruses in the Atacama Desert soils of the hyperarid core using read-based analyses of metagenomes; however, no information exists regarding host-virus interactions, dispersal, or the potential function of these viruses.

To understand the diversity and ecological impact of viruses inhabiting hyperarid soils, we investigated viral genomes assembled from soil metagenomes of the Atacama hyperarid core. We identified host-virus interactions, innate and adaptive host immunity elements, and phylogenetic diversity of viruses across geographically distant sampling locations. We analyzed putative extremotolerance genes and auxiliary metabolic genes (AMGs) found in the predicted viral sequences, providing evidence for a complex trade-off between viral predation and viral delivery of extremotolerance genes to microbes inhabiting harsh hyperarid desert soils.

## RESULTS

### Soil metagenomes of the Atacama hyperarid core feature heterogeneous viromes.

**FIG 1:**
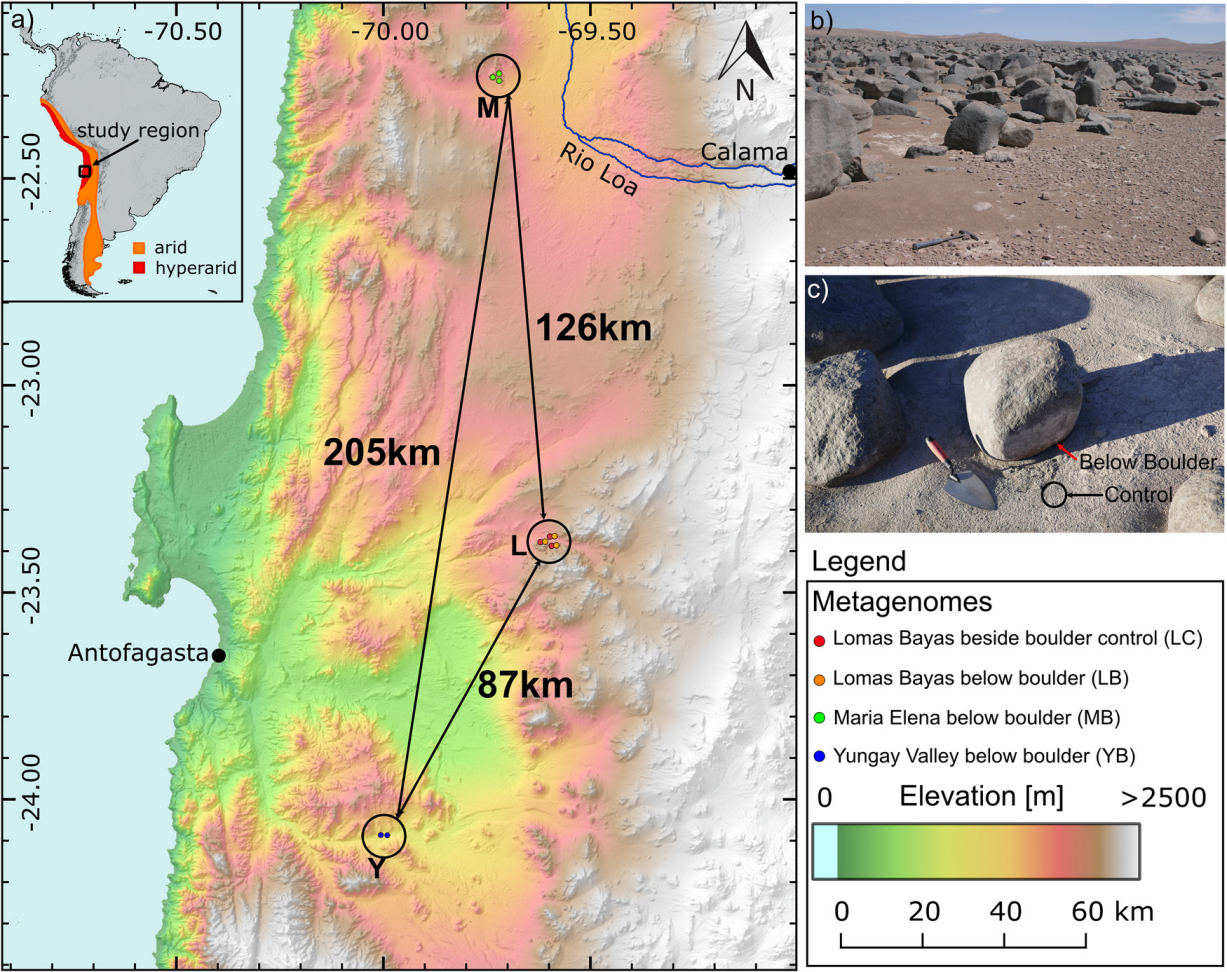
Sampling location information. (a) Map of the sampling locations M (Maria Elena), L (Lomas Bayas), and Y (Yungay Valley). Distances between sampling locations are shown. The number of metagenome samples per location is shown in circles. (b) Yungay boulder field. (c) A sampled boulder in Maria Elena boulder field. Red and black arrows indicate sample types B and C, respectively. The map was made using ASTER Global Digital Elevation Model (104).

We predicted viral scaffolds in 11 assembled metagenomes (4.1 Gbp in total) from three different boulder fields (Lomas Bayas [L], Maria Elena [M], and Yungay [Y]), which were previously studied for the impact of boulder cover on the soil microbiome, uncovering highly adapted microbes sheltered below the boulders of expansive boulder fields in the Atacama Desert hyperarid core (29). The aforementioned study compared the microbiomes in two different soil compartments (below the boulder [B] and control, i.e., exposed soil adjacent to the boulder [C]), for which we kept the designations consistent in this paper (for a map of the sampling locations, see Fig. 1; for the analysis workflow, see Fig. S1). In total, 6,809 of 707,509 examined scaffolds were predicted to be viral. After quality and length filtering, we identified 86 viral scaffolds (referred to here as viral genomes) with lengths of >0 kb forming 84 viral “populations” (dereplicated at 99% identity). In detail, VirSorter (35) predicted 79 viral genomes, while VIBRANT (36) predicted 37, including 30 overlapping between the two tools. The average length of the predicted viral genomes was 32.7 kbp (± 29.5 kbp), with the longest being 177 kbp and smallest being 10 kbp. The average G+C content of viral genomes was 58.7% (±10.3%), and the average coding density was 91.0% (±4.2%). The viral genomes were of varying quality: 9.30% “complete,” 10.5% “high quality,” 17.4% “medium quality,” 61.6% “low quality,” and 1.16% “not determined” according to CheckV (37). From these 84 viral populations, eight were predicted to be lysogenic. An overview of the viral population genomes can be found in Table S1a.

10.1128/mSystems.00385-21.1 TABLE S1 Supplemental information on viral population genomes, MAGs and sample geochemistry. (a) Overview of viral genomes, including their quality determined by CheckV (37), taxonomy information using vConTACT2 (87) and NCBI accession information. (b) Geochemistry and environmental data. Ion concentrations are measured in milligrams per gram of soil. (c) CRISPR-containing-bin info, repeat sequence, number of spacers, and Cas gene type. Identical repeat sequences are highlighted and color coded. (d) Locus information of putative extremotolerance genes and AMGs. (e) NCBI BioSample IDs and genome accession IDs for medium- to high-quality MAGs considered in this study. (f) Counts of different types of host antiphage system per MAG. Download Table S1, XLSX file, 0.4 MB. Copyright © 2021 Hwang et al. 2021 Hwang et al. https://creativecommons.org/licenses/by/4.0/ This content is distributed under the terms of the Creative Commons Attribution 4.0 International license.

10.1128/mSystems.00385-21.2 FIG S1 Flowchart outlining analyses carried out in this study. Software tools used are in italics, and resulting figures are in bold. Graphics are for illustrative purposes and not quantitative. Download FIG S1, TIF file, 0.8 MB. Copyright © 2021 Hwang et al. 2021 Hwang et al. https://creativecommons.org/licenses/by/4.0/ This content is distributed under the terms of the Creative Commons Attribution 4.0 International license.

**FIG 2:**
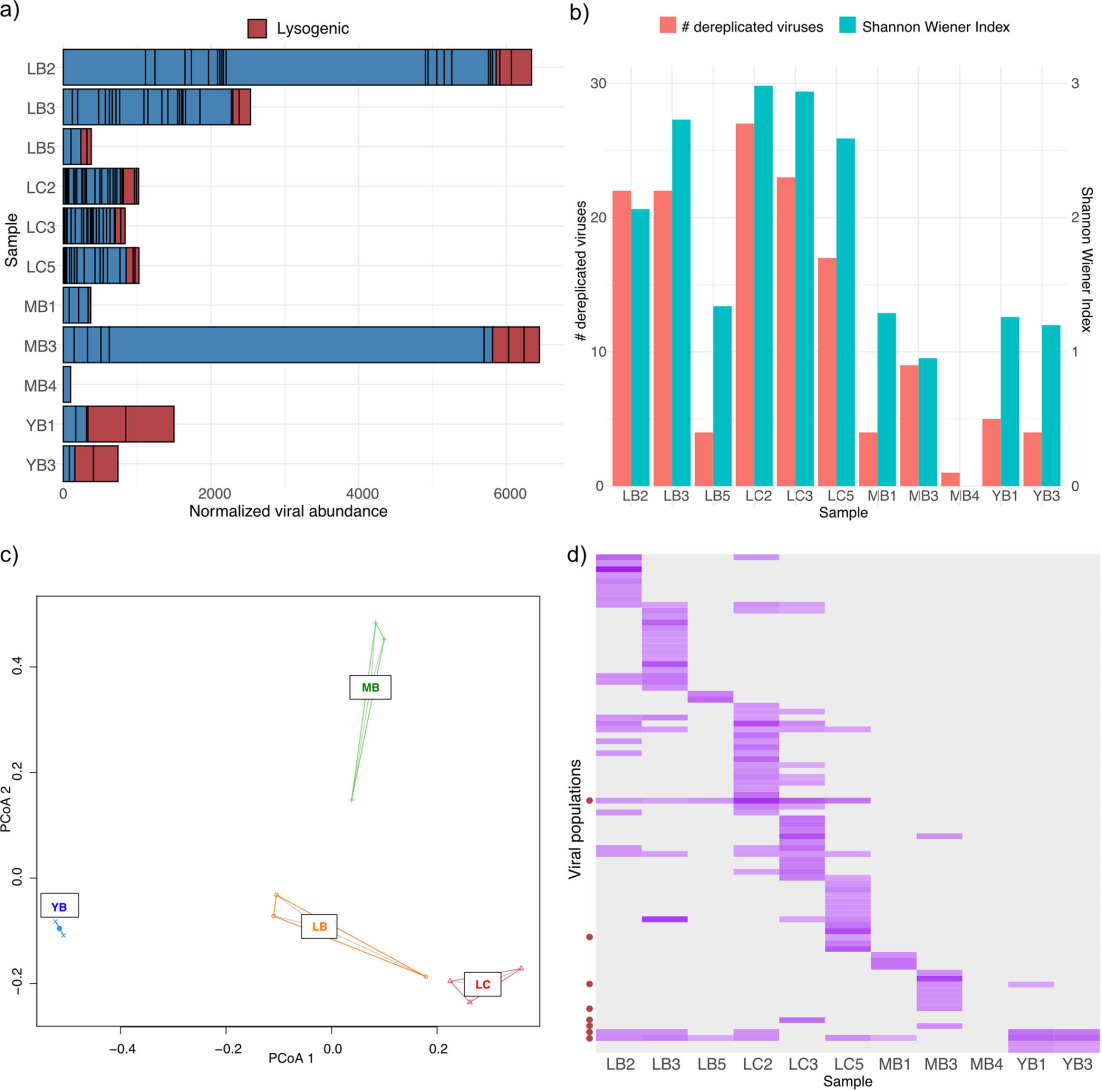
Abundance and diversity analyses of viromes. (a) Abundance of viral contigs normalized by sequencing depth of each sample. Each bar denotes a single viral population, and predicted lysogenic viral populations are marked in red. (b) Alpha diversity and species evenness of viromes. (c) PCoA plot of viromes using a Bray-Curtis distance matrix. (d) Relative abundance profile of each viral population. Darker purple denotes a viral population with higher relative abundance in a sample (gray indicates none detected). Lysogenic populations are marked with red circles on the *y* axis. Graphs were visualized in R v4.0.2 (81) using ggplot2 (83).

We observed a large degree of heterogeneity in the viral populations between samples, in terms of both alpha and beta diversity. Figure 2a shows the relative abundances of viral populations based on the total number of reads normalized across samples. The mapping-based coverage of viral populations identified in this study varied significantly, ranging between 4.5 and 5,075, and the total abundance of these viral populations varied up to 65-fold between samples. Notably, we did not detect prevalence of lysogenic viruses in L and M sites, while YB samples exhibited higher relative abundance (∼79%) of lysogenic viruses. Samples collected from L sites (*n* = 6) had higher alpha diversity and species evenness (Fig. 2b) than samples from M and Y (*n* = 5) (Welch’s *t* test, *P* = 0.0022 [alpha diversity] and *P* = 0.0056 [species evenness]). Principal-coordinate analysis (PCoA) (Fig. 2c) based on Bray-Curtis distances showed clustering by sampling site, while the beta diversities varied between sites, with YB viromes being particularly conserved and LB and MB viromes exhibiting higher variability than LC viromes. Permutational multivariate analysis of variance (PERMANOVA) (38) confirmed statistically significant differentiation of viral communities based on the sampling site (*R*^2^ = 0.384; *P* = 0.011). Biota-environment (BioENV) analysis (39) using geochemical and environmental metadata (Table S1b) revealed temperature and Na^+^ concentration to be most correlated with the viral community composition (rho = 0.5642; *P* = 0.001). The majority (66.7%) of the viral populations were unique to the sample; only one lysogenic virus with a representative genome of 12.6 kbp was detected across all three sites in eight samples, and three additional viral populations were observed between two different sites (Fig. 2d).

By comparing the distributions of viral and microbial genome abundances in each sample (Fig. S2), we found that only LC samples featured statistically significantly lower abundances (*P* < 0.05; Welch’s *t* test) of the viral genomes than microbial genomes, while no statistically significant differences could be observed in other samples. However, the ranges of viral genome abundances were greater than those of microbes in all samples, with samples LB2 and MB3 featuring viruses with abundances up to two orders of magnitude greater than those of microbes. We conducted symmetric co-correspondence analysis (sCoCA) to test whether the viral and microbial community compositions covary. The best sCoCA model using the first three axes determined the common variance between the viral and microbial communities to explain 37.2% and 56.8% of the total variances of viral and microbial communities, respectively (*P* = 0.006). The first three axes computed by sCoCA accounted for the 62.3% (CoCA1 = 26.5%; CoCA2 = 20.4%; CoCA3 = 15.3%) of the common variance. The first three axes of the ordinations in viral and microbial communities were highly correlated with each other (Pearson product-moment correlation coefficient > 0.99). The relative abundances of microbial taxa across samples used to conduct sCoCA are visualized in Fig. S3a, and the ordination biplots (Fig. S3b and c) illustrate highly similar positioning of the samples along the first two axes identified by sCoCA between microbial and viral communities. Higher species richness and species evenness (Shannon indices) could be observed in LB and LC samples for both microbial and viral communities; however, little pattern in the microbial population could be observed at the phylum level, suggesting that the covariance patterns are rooted in the abundance profiles of individual populations. Interestingly, neither viral nor microbial communities were predictive of each other when CoCA was conducted in predictive mode (*P* > 0.05) (40).

10.1128/mSystems.00385-21.3 FIG S2 Normalized abundances of microbes (M) and viruses (V). Quality-filtered reads were mapped to viral scaffolds and ribosomal protein S3 gene (*rpS3*)-containing scaffolds for abundance estimation of viral and microbial population genomes, respectively. Coverages were normalized by the sequencing depth of each sample. Visualization was done in R v4.0.2 (81). Significant differences (*P* < 0.05; Welch’s *t* test) between the microbial and viral abundances in a sample are marked with an asterisk. Download FIG S2, TIF file, 0.2 MB. Copyright © 2021 Hwang et al. 2021 Hwang et al. https://creativecommons.org/licenses/by/4.0/ This content is distributed under the terms of the Creative Commons Attribution 4.0 International license.

10.1128/mSystems.00385-21.4 FIG S3 Microbial community compositions and their covariation with viral communities. (a) Relative abundance profiles of microbial communities. Bars represent distinct microbial population and are color coded according to phylum-level taxonomic classification. (b and c) Co-correspondence ordination biplot. Samples are indicated with empty diamonds, viral populations with crosses (b), and microbial populations with filled circles (c). Colors used to represent the phyla of microbial populations are identical to those in the legend in panel a. The mesh of the grid is indicated with the value “d.” FIG S3, TIF file, 1.9 MB Copyright © 2021 Hwang et al. 2021 Hwang et al. https://creativecommons.org/licenses/by/4.0/ This content is distributed under the terms of the Creative Commons Attribution 4.0 International license.

### Atacama viruses are phylogenetically novel and diverse.

**FIG 3:**
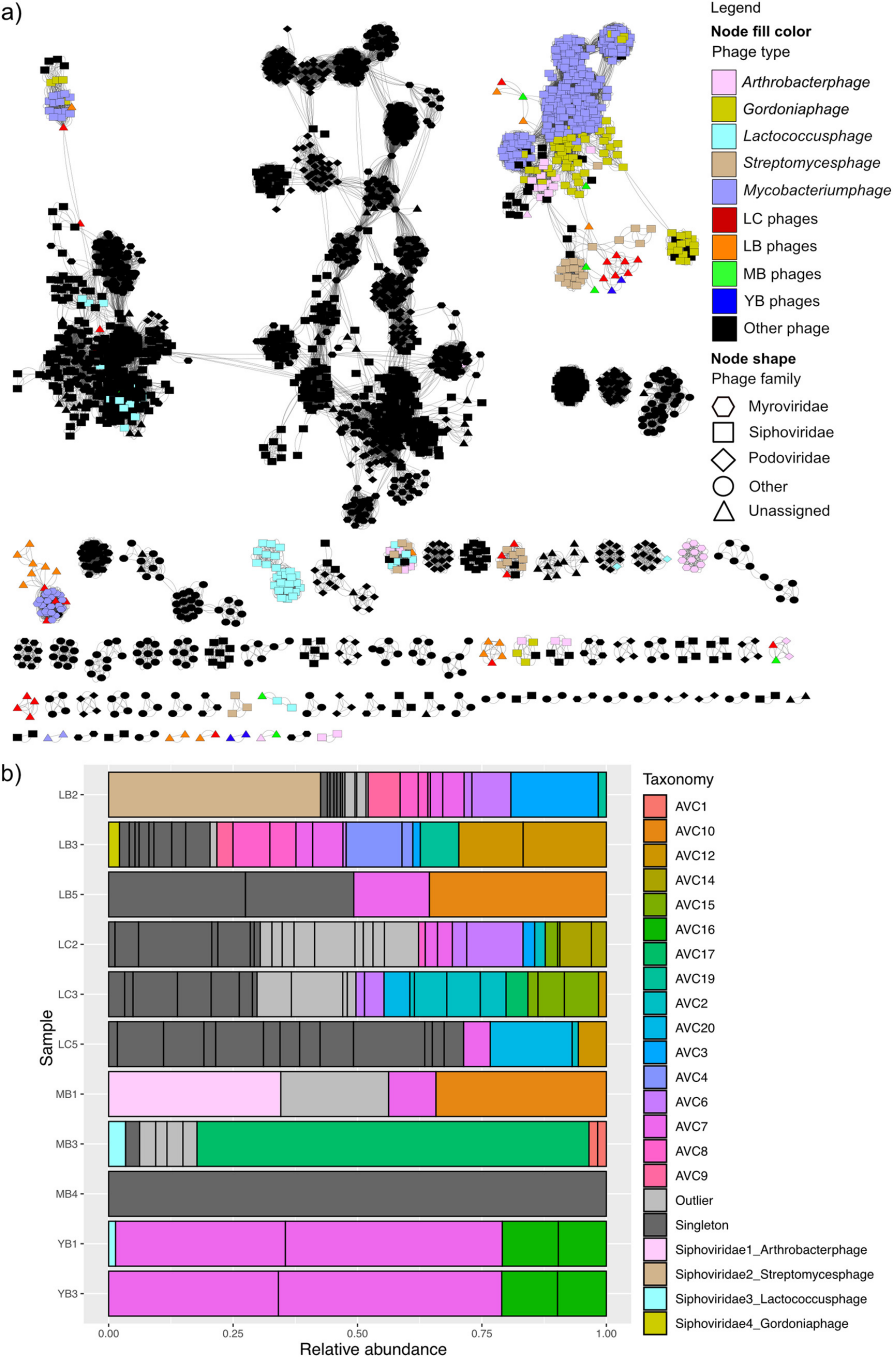
Clustering of Atacama viral genomes with reference viral genomes based on shared genes. (a) vConTACT2 output network with a significance greater than 1. The network was visualized using Cytoscape v3.8.0 (88). Queried viruses are color coded based on the sampling site they were recovered from, and reference viruses are color coded based on the host they were annotated to infect. Node shape denotes the phage family of reference viruses. (b) Relative abundance of identified taxonomic groups per sample. AVC denotes genus-level Atacama viral clusters identified using vConTACT2. Each bar represents a distinct viral population.

We clustered 84 dereplicated viral population genomes using intergenomic similarities (41). Eighty-two clusters were formed at the genus level (intergenomic similarity threshold at 70%), indicating that all except two viral population genomes recovered in this study are of different genera. The OPTSIL clustering of viruses (42) yielded 84, 64, and 14 clusters at the species, genus, and family levels (Fig. S4). vConTACT2 (43) was used to cluster the Atacama viral genomes with 2,616 known prokaryotic viruses (Fig. 3a). Twenty-two Atacama viral genomes were related to phages infecting *Gordonia*, Mycobacterium, *Streptomyces*, and *Arthrobacter* at a taxonomic level higher than genus. Only four viral contigs were predicted to be in the same genus as a *Gordonia* phage, a *Streptomyces* phage, a *Lactococcus* phage, and an *Arthrobacter* phage from the reference database, all belonging to the order *Caudovirales*, family *Siphoviridae* (tailed double-stranded-DNA [dsDNA] phage). vConTACT2 also predicted 20 genus-level clusters consisting of two to five Atacama viruses (Atacama viral clusters [AVC]). Of the remaining viral populations that could not be clustered, 27 were classified as “singleton” viruses, which share no or very few genes with the database and each other, and 19 “outlier” viruses, which could be associated with existing sequences but could not be clustered due to a low confidence level. Atacama viral genomes tended to cluster with each other in the gene sharing network rather than with the viral sequences in the database. Notably, the majority of the Atacama viruses that are related to reference *Streptomyces* phages and Mycobacterium phage were recovered from the LC samples. Across the three different tools, we estimated between 64 and 80 genus-level clusters among the 84 viral populations.

10.1128/mSystems.00385-21.5 FIG S4 Phylogenomic genome BLAST distance phylogeny (GBDP) trees by VICTOR. Trees were inferred using the formula D6 with nucleotide sequences of 86 viral genomes as the input, yielding average support of 86% and 78%, respectively. The numbers above branches are GBDP pseudo-bootstrap support values from 100 replications. The branch lengths of the resulting VICTOR (41) trees are scaled in terms of the respective distance formula used. Virus genomes are color coded according to the sampling site the genomes were recovered from. Red, LC; orange, LB; green, MB; blue, YB. Download FIG S4, TIF file, 1.2 MB. Copyright © 2021 Hwang et al. 2021 Hwang et al. https://creativecommons.org/licenses/by/4.0/ This content is distributed under the terms of the Creative Commons Attribution 4.0 International license.

The relative abundance profiles of different taxonomic groups in each sample (Fig. 3b) illustrated a high level of heterogeneity between the three sites as well as among the samples collected from sites L and M. In contrast, the two Y samples were almost identical in the taxonomic composition of their viromes. For all the samples, singletons and outlier clusters of viral genomes that are biologically novel (due to very little protein homology to the existing database) constituted the majority.

### Sequence-informed putative host-virus interactions indicate dispersal of hosts and/or viruses.

**FIG 4:**
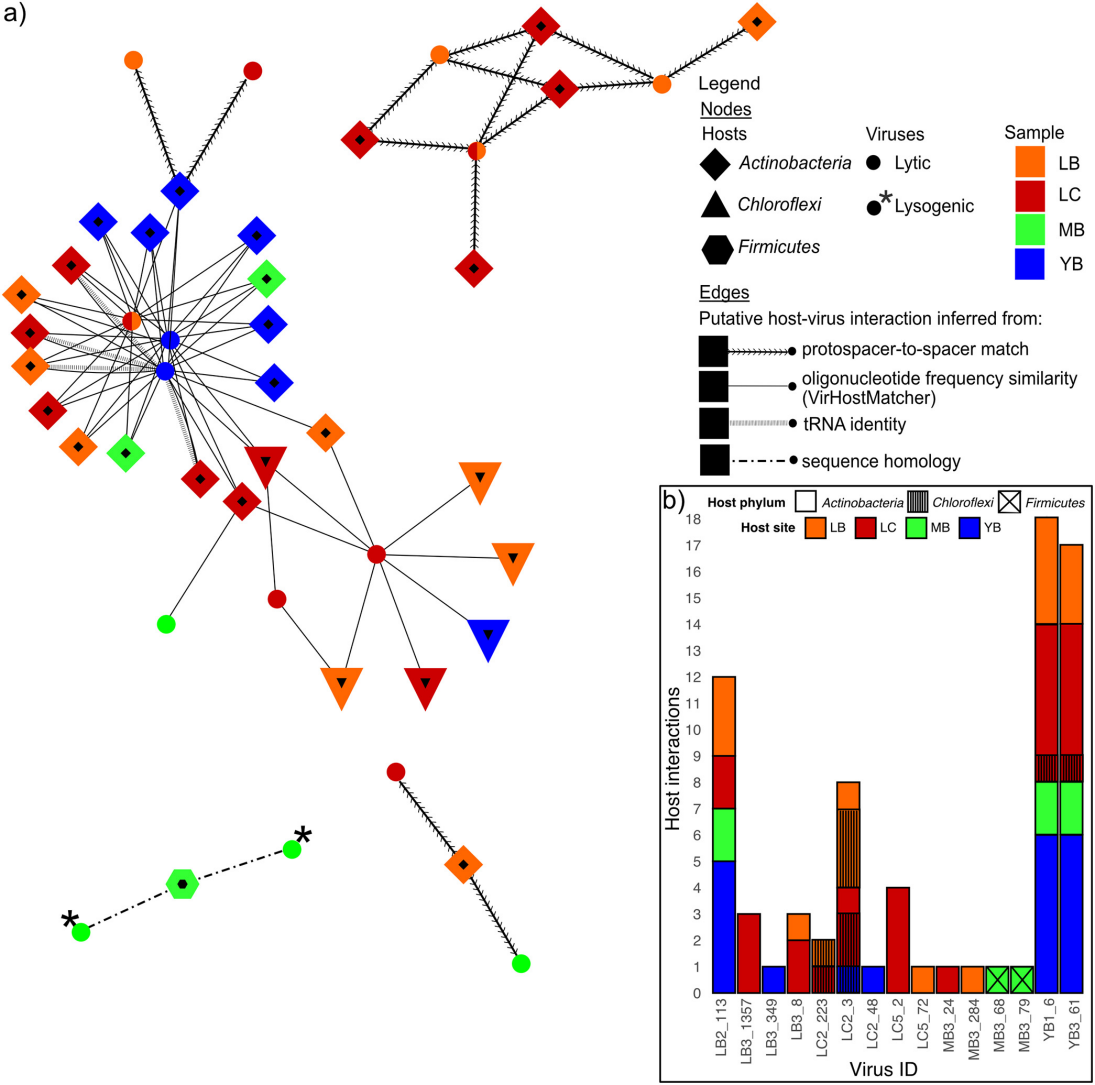
Host-virus interactions. (a) Putative host-virus interaction network displaying host taxonomy, host location, virus location (if a virus was detected in multiple samples, multiple colors are used), and virus lifestyle. Edges denote the method used to predict the host-virus interaction. Visualization was done using Cytoscape v3.8.0 (88). (b) Number of host interactions per virus, where the bar color denotes the site from which the matched MAGs were assembled and bar texture denotes host taxonomy.

Previously, we recovered 73 medium- to high-quality (>75% completeness; <15% contamination) metagenome-assembled genomes (MAGs) across 11 metagenomes from three sampling locations (29). The MAGs were classified as 34 *Actinobacteria*, 30 *Chloroflexi*, eight *Thaumarchaeota*, and one *Firmicutes*. We resolved 74 unique interactions between 30 MAGs and 15 viruses using the following four sequence-based methods: (i) protospacer-to-spacer match for MAGs containing CRISPR arrays, (ii) oligonucleotide frequency similarity (VirHostMatcher) (44), (iii) tRNA matching, and (iv) nucleotide sequence homology. Figure 4a illustrates the putative host-virus interactions, specifying the method by which the interactions were resolved.

We identified 14 unique interactions between six actinobacterial MAGs with CRISPRs and seven viruses. Of 73 MAGs surveyed, nine actinobacterial and two *Chloroflexi*-derived MAGs contained CRISPR arrays from which direct repeat (DR) sequences were extracted. All MAGs carried unique sets of DR sequences, although three identical DRs appeared across six MAGs (see Table S1c for details). In total, 18 identified DR sequences were used to recover 3,438 unique spacers directly from the reads in their respective metagenomes. Five actinobacterial MAGs classified as *Rubrobacter* recovered from site L clustered based on their shared infection histories with three phylogenetically distinct viruses also detected from site L, assuming that the CRISPR arrays were not horizontally transferred (45). Interestingly, two additional actinobacterial MAGs (one *Rubrobacter* and one *Acidimicrobiia*) had acquired spacers that results in resistance against viruses detected from sites between 87 and 205 km away (Fig. 2), which indicates viral and/or host dispersal across the desert. In sum, spacer-to-protospacer-based identification of host-virus interactions revealed potentially widely dispersed viruses preying on *Actinobacteria*, particularly *Rubrobacter*.

Although the spacer-to-protospacer matches between CRISPR-containing MAGs and viral genomes provide high-confidence evidence of historical infections between a host population and viruses, many bacteria and archaea do not have CRISPR-Cas defense systems (46) or the respective CRISPR arrays do not get assembled or binned into MAGs. In the metagenomes studied, only 17% of the medium- to high-quality MAGs contained CRISPR arrays. To predict possible host-virus interactions for hosts that lack CRISPR systems, VirHostMatcher (44) identified 54 putative interactions between 23 MAGs (17 *Actinobacteria* and six *Chloroflexi*) and six viral genomes based on shared k-mer frequency patterns. Most linkages were established between 16 actinobacterial MAGs belonging to class *Acidimicrobiia* and three viruses belonging to two genus-level clusters. Interestingly, VirHostMatcher matched some viruses to hosts that are taxonomically distant, some differing at the order level and a few even in different phyla. For instance, three viruses were matched to both *Chloroflexi* and *Actinobacteria* (Fig. 4b), suggesting the possibility that these viruses have broad host ranges. No overlaps between interactions based on spacer-to-protospacer matches and VirHostMatcher host-virus linkage were identified.

Additionally, tRNA matching and a sequence homology search were conducted. We detected between 1 and 35 tRNAs across 20 viral population genomes. Only complete identity between viral and host tRNAs was used as an indication of potential host-virus interaction. We identified four interactions between four actinobacterial MAGs and one virus detected from the Y site. These interactions overlapped interactions inferred from oligonucleotide frequency similarity. Finally, nucleotide sequence homology was used to identify putative host-virus interactions between two lysogenic viruses and a firmicutal MAG. Figure 4b summarizes the putative host-virus linkages for each viral genome, visualizing a high degree of variance in the number of identified interactions per virus, as well as the evidence of putative cross-site host-virus interactions and the potential for broad host ranges for some viruses.

### Atacama viruses carry genes against environmental stress.

**FIG 5:**
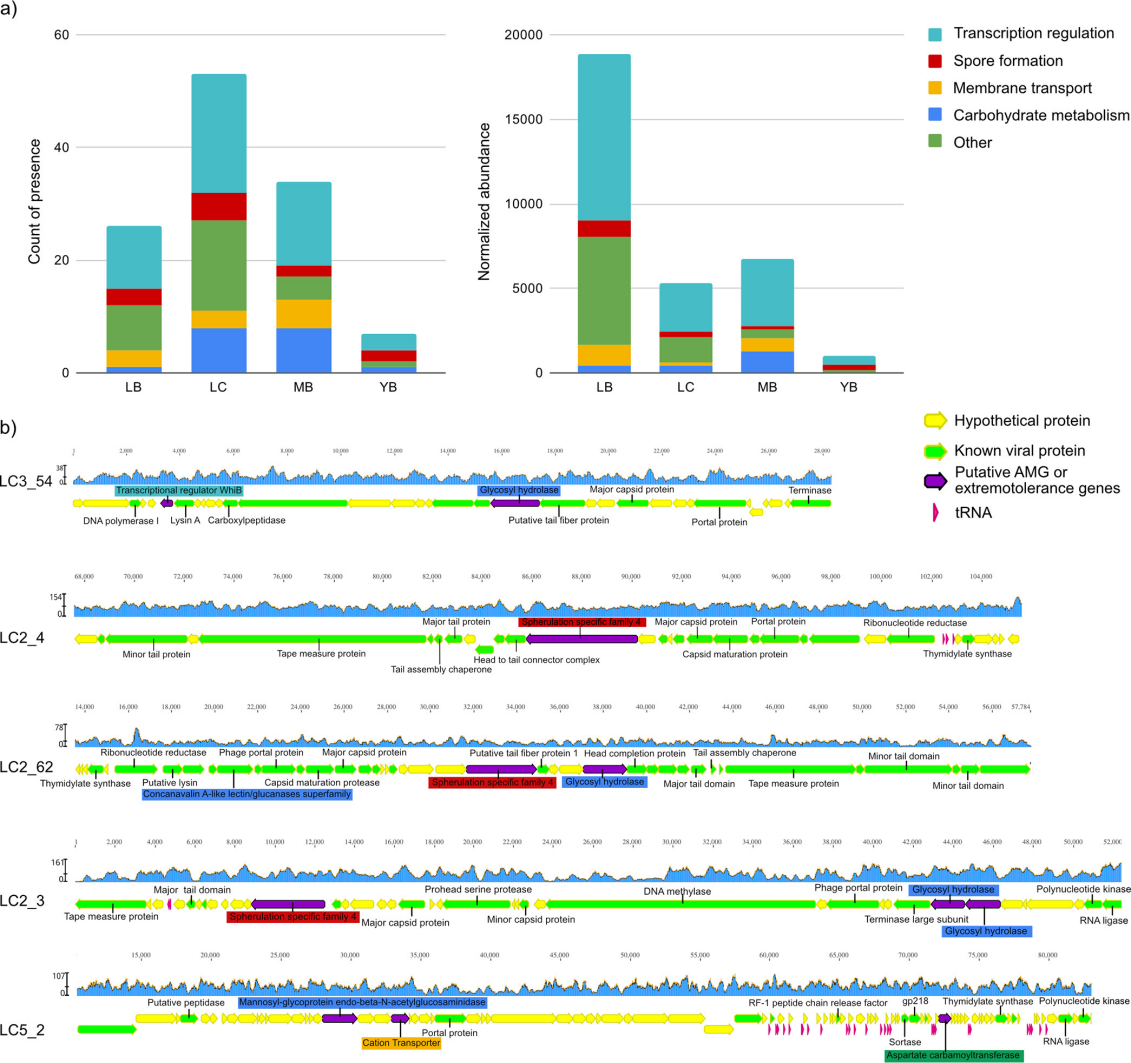
Extremotolerance and auxiliary metabolic genes. (a) Sums of counts of presence (left) and normalized abundance (right) of putative extremotolerance genes and AMGs across sampling sites. Abundances were calculated using the coverage of scaffolds where the gene was found and normalized across samples by sequencing depth. (b) Genomic neighborhoods of putative extremotolerance genes and AMGs. Genes of interest are colored purple, and labels are color coded by the functional categories according to the color scheme used in panel a. Identified viral genes are labeled and colored green, while gene homologs to a viral protein in the DRAM-v (47) database without a known function are colored green without a label. Genes of uncharacterized function and tRNAs are in yellow and pink, respectively. Coverages calculated based on mapped reads are shown in the blue graph above each genomic region visualized with Geneious v11.1.5.

Across 84 viral populations, we identified and annotated 4,288 proteins using DRAM-v (47). Thirty-nine percent of these proteins could be associated with the sequences in the queried database with high confidence, of which approximately half were “uncharacterized” and “hypothetical” proteins. We found 58 genes likely involved in extremotolerance and/or metabolism of microorganisms (for locus information, see Table S1d) across 30 viral populations. For instance, we found a sporulation protein (spherulation-specific family 4 [SSF] protein) in three viral genomes assembled from the LC2 sample. Some viruses also encoded membrane transport proteins for cation transporters and potassium channels, while others encoded transcriptional factors, including WhiB (48), which is also involved in bacterial sporulation initiation (49). Other putative AMGs included PhoD-like phosphatase, esterase, glucanases, glycosyl hydrolases, and endo-beta-*N*-acetylglucosaminidase. Interestingly, we also identified LuxR among the AMGs, the response regulator involved in quorum sensing of bacteria, which binds homoserine lactones and activates genes in the respective operon (50). Putative extremotolerance genes and AMGs were present across all sampling sites (Fig. 5a), with the LB site exhibiting the highest abundance of viruses carrying these genes, while the LC site harboring the highest diversity of these genes. Figure 5b visualizes genomic regions of example scaffolds carrying genes of interest, displaying relatively uniform coverage across all gene loci and a close vicinity of viral hallmark genes, providing direct evidence that these genes are likely bona fide features of these viral genomes.

We further investigated the evolutionary relationship of viral genes relative to host homologs for two genes of interest: SSF genes and *whiB*. These two genes are generally known to be associated with bacterial spore formation and therefore could provide key extremotolerance for microbes in harsh desert environments. We compared the three genomic regions containing the SSF genes (Fig. S5a) and found that the genes upstream and downstream of the SSF genes in the viral scaffolds LC2_4 and LC2_62 showed synteny despite the lack of sequence homology between the SSF proteins themselves. On the other hand, SSF proteins found in LC2_62 and LC2_3 contained homologous regions, despite the lack of synteny and the disparity in the GC content throughout the genome. We examined the evolutionary history of these SSF proteins by placing them on a phylogenetic tree with similar bacterial proteins deposited in the NCBI nr database (Fig. S5b) and 10 SSF protein homologs found across nine MAGs from this environment. Interestingly, only actinobacterial MAGs binned from the L site metagenomes contained this gene and formed two distinct clusters. Compared to publicly available SSF proteins, the viral SSF proteins identified herein were not closely related to their homologs found in reconstructed MAGs. The closest homologs to the viral SSF proteins were found in genomes of *Actinobacteria* (e.g., *Streptomyces*), which contains many spore-forming bacteria. Notably, the viral genome of LC2_3 was predicted to have interactions with *Actinobacteria* and *Chloroflexi* in our samples (based on oligonucleotide frequency similarity) (Fig. 4), and two actinobacterial MAGs also encoded distantly related SSF proteins (predicted virus-MAG interactions are shown with red lines in Fig. S5b). Based on the evidence of unique SSF proteins found in three distinct viral genomes and the phylogenetic divergence from host proteins, the likelihood that they are randomly packaged host genes is low. Although the function of the viral SSF proteins requires experimental confirmation, we suspect that this relatively large gene (mean protein length = 1,265 amino acids) is beneficial for the viral populations, as it was identified in multiple viruses despite the presumed high cost of maintenance of AMGs in viral genomes (51, 52).

10.1128/mSystems.00385-21.6 FIG S5 Analysis of spherulation-specific genes and proteins regarding their genomic neighborhood and phylogenetic placement relative to host genes. (a) To identify potential synteny between viruses carrying spherulation-specific family (SSF) genes, we blasted SSF gene-containing regions against each other (E value threshold, 1E−5) and visualized them using Easyfig 2.2.2 (94). The results depict synteny between LC2_4 and LC2_62 regarding multiple genes and their arrangements, but only LC2_62 shared the gene of interest with LC2_3. GC content along the regions is visualized directly above the genome maps. Putative promoters and terminators are indicated using a red star and triangle, respectively. Purple genes denote identified extremotolerance genes or AMGs, green genes are viral according to significant homology with the viral protein database, and yellow genes are hypothetical or uncharacterized genes. (b) Phylogenetic placement of the three viral spherulation-specific protein sequences with their highest-identity BLASTp hits in the NCBI’s nr database. The tree was calculated via MUSCLE alignment (95) of the protein sequences followed by BMGE v1.12 trimming (96) using a BLOSUM62 matrix, and iqtree v1.5.5 (97) with the flags -m MFP -alrt 1000 -bb 1000. The tree was visualized using iToL (98), and branches marked with black circles are considered strongly supported (SH-alrt test value > 80; bootstrap value > 95). Red lines denote predicted host-virus interactions between the viral contig and bacterial MAG containing the spherulation-specific proteins. Download FIG S5, TIF file, 1.7 MB. Copyright © 2021 Hwang et al. 2021 Hwang et al. https://creativecommons.org/licenses/by/4.0/ This content is distributed under the terms of the Creative Commons Attribution 4.0 International license.

A WhiB-like transcription factor has previously been shown to control the spherulation septation in *Actinobacteria* (49). In addition, WhiB-like proteins previously identified in several Mycobacterium phages and *Streptomyces* phages (53) have been shown to regulate host cell wall component alteration in mycobacteria (54). Twelve WhiB-like transcription factor genes were identified across 11 viral populations as well as in 49 actinobacterial MAGs across all three sites. We phylogenetically placed the 12 viral WhiB-like proteins with bacterial WhiB proteins found in medium- to high-quality MAGs (Fig. S6a). Viral WhiB-like proteins were generally more related to each other than to the bacterial proteins. Additionally, WhiB-like proteins found in interacting virus-microbe pairs (Fig. S6a) were not closely related compared to other homologs. Notably, viral WhiB-like proteins from the same site tend to cluster together phylogenetically, with the exception of the LC samples. When comparing these proteins with related sequences in the NCBI nr database, we found four clusters with phage proteins, while the rest (eight proteins) clustered with homologs found in bacteria (Fig. S6b).

10.1128/mSystems.00385-21.7 FIG S6 Phylogenetic analyses of viral WhiB-like proteins. (a) Phylogenetic placement of viral WhiB-like proteins and microbial WhiB-like proteins identified in the same metagenomes. (b) Phylogenetic placement of viral WhiB-like protein sequences and their highest-identity blastp hits in the NCBI nr database. The tree was calculated via MUSCLE alignment (95) of the protein sequences followed by iqtree v.1.5.5 (97) with the flags -m MFP -alrt 1000 -bb 1000. The tree was visualized using iToL (98), and branches marked with black circles are considered strongly supported (SH-alrt test value > 80; bootstrap value > 95). Queried sequences are color coded according to the sampling site. Orange, LB; red, LC; green, MB; blue, YB. Download FIG S6, TIF file, 1.4 MB. Copyright © 2021 Hwang et al. 2021 Hwang et al. https://creativecommons.org/licenses/by/4.0/ This content is distributed under the terms of the Creative Commons Attribution 4.0 International license.

## DISCUSSION

The hyperarid core of the Atacama Desert harbors an abundant and diverse soil virome that has been mostly understudied due to the scarcity of microbial biomass available. Recent improvements in soil DNA isolation methods and deeper sequencing of metagenomes (28, 29) not only allowed the discovery of microbes actively replicating *in situ* but also shed light upon the viral fraction of the hyperarid soil ecosystem that coexists with their microbial hosts. Our investigation of the Atacama viromes reveals taxonomically diverse viruses and complex interactions between viruses and their hosts across the desert. Notably, the viruses contained key extremotolerance genes, and we propose a mutualistic model of host-virus interaction, where viruses seek protection in microbes as lysogens and pseudolysogens and, in return, aid host extremotolerance and survival.

### Diversity of Atacama viruses contradicts the scarcity of microbial hosts.

The Atacama Desert soil virome analyzed in this study consists of 84 viral populations belonging to at least 60 novel genera. The diversity of the viruses in the Atacama Desert soils is astonishing considering that the microorganisms that inhabit these soils are low in both biodiversity and biomass. Typically, groups of viruses infecting the same host often exchange genetic materials and form genotypic clusters (55). Therefore, in a low-diversity ecosystem, where many viruses infect the same hosts, one may expect a stronger genotypic clustering of viruses. Additionally, a large diversity of viral predators coupled with low diversity of prokaryotic prey seems to go against the competitive exclusion principle (56). High abundance and diversity of viruses in an environment with reduced encounters with viable microbial hosts suggest that some of the Atacama soil viruses may be dormant virions waiting for the appropriate host population to thrive, while others remain protected by residing in the host cells as lysogens (integrated into host genomes or plasmids) or pseudolysogens (as virus particles in the host cytoplasm) (24, 57). In particular, viruses have been shown to seek protection in their host cells, and this mode of viral survival has been observed in hot hyperarid desert soils, where lysogenic and pseudolysogenic [referred to together here as (pseudo)lysogenic] phages were found to be more prevalent (24–26). In our study, we predicted eight viral “populations” to be lysogenic based on their representative genomes being proviral. However, computational prediction tools tend to underestimate lysogenic viruses (35) and cannot distinguish pseudolysogens from lytic viruses. Therefore, isolation, cultivation, and visualization of the viruses and their hosts would shed light on the lifestyle of the viruses in the Atacama Desert hyperarid soils once sufficient biomass can be harvested from the ecosystem.

### Viral dispersal is likely host mediated across the Atacama Desert.

Our study demonstrated high co-correspondence between the viral and microbial communities, possibly due to the sample- and site-specific environmental stressors controlling both microbial and virus populations. Despite the high heterogeneity and specificity between samples, we identified four viral populations (two of which were lysogenic) detected in two or more sites (L, M, and Y). None of these genomes could be linked to a host, while 35 of 74 predicted host-virus interactions were between microbes and viruses detected at different sites. In particular, four host-virus interactions based on protospacer-to-spacer matches provide evidence for past dispersal events of either microbial and/or viral populations across distances up to 205 km. We hypothesize that one specific mechanism of dispersal in the Atacama Desert is the frequent sandstorms and powerful winds (58, 59) transporting infected microbes and virions in organic aerosols (60, 61). Notably, the most frequently detected viral population across samples (in 8 of 11 samples) was predicted to be lysogenic, supporting the scenario of viral dispersal through the transport of infected hosts. Additionally, we observed statistically significantly lower abundance of viral entities relative to microbes (Fig. 5S) in C samples, suggesting that viruses are vulnerable to irradiation in desert environments, perhaps more so than microbes. This result may also indicate that viral entities sheltered below boulders undergo lytic cycles and therefore exhibit higher abundance than microbes, while those beside boulders are primarily (pseudo)lysogenic. Further work is required to reveal the predominant lifestyle and viability of viruses in the Atacama hyperarid core.

### A mutualistic model of host virus interactions in the Atacama Desert hyperarid core.

A closer look at the genes carried by the Atacama viruses suggested an intriguing interaction between viruses and hosts, where a fine balance between viral predation and host extremotolerance sustains the continuum of the ecosystem. We posit that the viruses may serve as vectors delivering extremotolerance genes to their microbial hosts, increasing the chance of microbial survival under the harsh conditions of the Atacama Desert hyperarid core. In particular, we propose a model specific to extreme deserts, where (pseudo)lysogenic viruses encoding extremotolerance genes could support microbial survival in exchange for taking up shelter inside the microbial cytoplasm or genome. This mutualistic model closely parallels viral AMGs found in temperate environments (e.g., photosystem I and II genes in marine cyanophages [62–64] and CAZymes in mangrove soil viruses [65]), where AMGs are selected to maximize viral production by enhancing host metabolism during an infection. In the hyperarid core of the Atacama, viral extremotolerance genes likely increase the chance of viral production by aiding host survival, even if they result in temporary dormancy of the host through sporulation (in the case of SSF protein and WhiB).

A similar model of virus-host interactions (66) has been described in biofilms, where lysogenic viruses support formation, stabilization, and dispersal of biofilms, and biofilms in return provide protection for viruses against environmental stress (67–69). For instance, in hot desert soils, where microbes are known to form biofilms to protect themselves from desiccation, UV radiation, and low nutrient availability (70), Zablocki et al. (21) hypothesized a positive selection for temperate viruses in desert biofilms.

**FIG 6:**
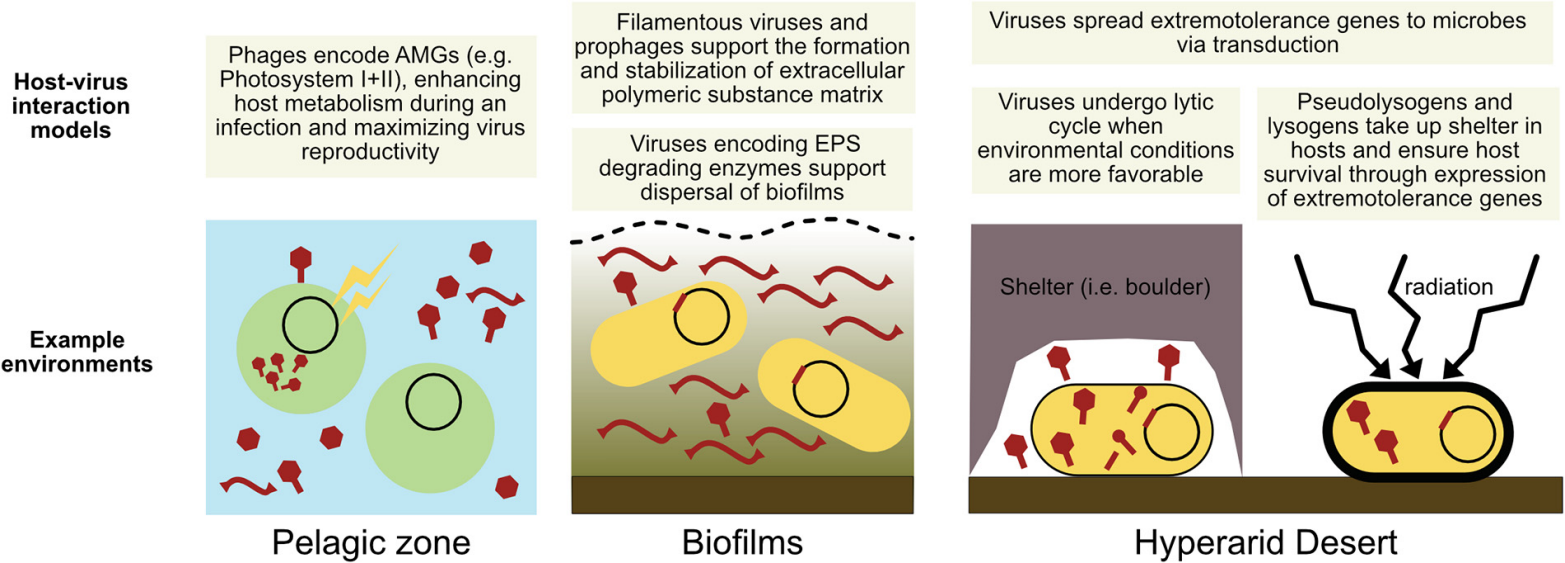
Visual schematic of the proposed host-virus interaction models in the hyperarid core of the Atacama Desert and comparison with models in other environments. Viruses (virions, pseudolysogens, and prophages) are shown in dark red, and microbial cells are in yellow and green.

In even more extreme desert environments, such as the hyperarid core of the Atacama, where we did not detect any formation of biofilms, viruses may instead seek protection in the cytoplasm of the microbes as (pseudo)lysogens, as previously identified in other hot deserts (24–26). In this case, viral genes encoding extremotolerance may be selected for two reasons: (i) to ensure the survival of both the microbe and the (pseudo)lysogenic virus in the short term and (ii) to spread extremotolerance genes among microbes via transduction or lysogeny, resulting in the long-term increased fitness of the hosts against environmental stress. A visual schematic of the proposed host-virus interactions in the Atacama hyperarid core and a comparison with proposed host-virus interactions in other environments are found in Fig. 6. This mutualistic model does not exclude the antagonistic interactions between viruses and their hosts, as evidenced by the diverse innate and adaptive antiphage systems encoded in host MAGs (for an analysis of innate and adaptive immune systems, see Text S1).

10.1128/mSystems.00385-21.8 TEXT S1 Preliminary analysis of innate antiphage defense systems in MAGs. Download Text S1, DOCX file, 0.02 MB. Copyright © 2021 Hwang et al. 2021 Hwang et al. https://creativecommons.org/licenses/by/4.0/ This content is distributed under the terms of the Creative Commons Attribution 4.0 International license.

We posit that viruses undergo lytic cycles when more favorable environmental conditions are met (i.e., rain events) or even in sheltered environments, resulting in the observed higher relative viral abundances under boulders compared to beside boulders. Phylogenetic analyses of the viral homologs to SSF proteins and the WhiB-like transcription factors suggest that these genes are indeed distinct from bacterial homologs, including those found in putative hosts. We also observed phylogenetic clustering of viral homologs to WhiB-like transcription factors by sampling site, suggesting the possibility of individual adaptation to the host communities and/or the environmental conditions of each sampling site. Model host-virus systems will be required to confirm our proposed model (Fig. 6) and identify to what extent extremotolerance genes provide an increase in fitness for microbes and viruses in hyperarid environments.

### Conclusion.

The hyperarid core of the Atacama Desert is a much more biologically complex ecosystem than previously thought. We investigated hyperarid soil metagenomes to uncover a diverse virome interacting with a wide range of microbial hosts. Viruses in the Atacama Desert not only endure long periods of desiccation and extreme oxidative stress themselves but may also deliver extremotolerance genes to their hosts and aid their survival. This study expands the ecological significance of viruses in terrestrial systems, particularly in deserts. Life seems to persist even in the most hostile environments on Earth, and so do viruses. The Atacama Desert virome and its complex interplay with extremotolerant host populations highlight the role viruses play in microbial evolution and dynamics and illustrate a new dimension to host-virus interactions in extreme environments.

## MATERIALS AND METHODS

### Sampling location, procedure, and metagenomic library preparation.

Briefly, sampling was conducted in March 2019. Three sampling sites, Yungay (Y), Maria Elena (M), and Lomas Bayas (L), were chosen from the hyperarid core of the Atacama Desert (a map of the sampling locations is shown in Fig. 1). Samples were collected from below boulders (B) and in the exposed surface soil (control [C]) beside boulders. Three B samples (LB2, LB3, and LB5) were collected at the Lomas Bayas boulder field, three B samples (MB1, MB3, and MB4) were from the Maria Elena boulder field, and two B samples (YB1 and YB3) were from the Yungay boulder field. Each B sample was collected from soil below one unique boulder, where the number in the sample name corresponds to the specific boulder. Three C samples (LC2, LC3, and LC5) were taken from the Lomas Bayas boulder field, from the exposed surface soil beside corresponding sampled boulders. Eleven metagenomic libraries of DNA extracted from eight B samples and three C samples were sequenced on Illumina HiSeq 2500 (Illumina, California, USA). Details of the sampling procedure, site coordinates, DNA extraction, and Illumina library preparation and sequencing can be found in our previous study (29).

### Metagenomic analysis, host genome binning, and taxonomic classification.

Descriptions of the assembly of metagenomic reads, contig binning, and bin analyses can be found in our previous study (29). Only medium- to high-quality bins (>75% completeness and <15% contamination calculated using CheckM v1.0.13 [71]) were considered host genomes. Host taxonomy was predicted using GTDB-Tk classify_wf (72). MAGs underwent gene prediction using Prodigal v2.6.3 (73) in meta mode and were annotated using Diamond v0.9.9 (74) against the UniRef100 database (75). The annotations were subsequently screened for identification of host innate defense marker genes identified by Bezuidt et al. (76).

### Prediction and analysis of viral scaffolds.

A schematic illustration of the analyses conducted can be found in Fig. S1. VirSorter v1 (35) with default settings and the –diamond flag, as well as VIBRANT v1.2.1 with default settings (36), was used for viral signal prediction across all assembled metagenomes and scaffolds length of ≥1,000 bp. VirSorter-predicted viral scaffolds in categories 1 and 2 were combined with VIBRANT-predicted viral scaffolds with qualities of medium, high, and complete. A viral contig was considered lysogenic if it was predicted as such by at least one of the following tools: VIBRANT, VirSorter, and CheckV v0.6.0 (37). Similarly, a virus was considered complete if at least one of the three previously mentioned tools predicted it to be circular or complete. Viral contigs were dereplicated using CD-HIT v4.6 (77) at 99% identity to identify viral populations, which were used for all subsequent analyses. CheckV v0.6.0 was used for completeness and quality estimation. Abundances of viral genomes were estimated using coverage calculated across all samples using a method described by Roux et al. (78). In short, reads were mapped using Bowtie2 (79) at ≥90% identity using the options –ignore-quals –mp = 1,1 –np = 1 –rdg = 0,1 –rfg = 0,1 –score-min = L,0,-0.1 as suggested by Nilsson et al. (80). Coverages were calculated only for scaffolds with mapped reads across ≥75% of the scaffold length with ≥1× coverage, for which average per-base coverage was calculated. Abundances of MAGs were estimated using the coverage of the ribosomal protein S3 (rpS3)-containing scaffold as described in our previous study (29). Calculated coverages were then subsequently normalized across samples by the total number of reads per sequenced library, to control for the sequencing depth of each sample. Statistically significant differences between viral and microbial abundance per sample were determined using Welch’s *t* test with a *P* value threshold of <0.05. Viral genome annotation was performed using DRAM-v (47) using the UniRef90 database (75) and all other default databases in DRAM without the KEGG annotations.

### Statistical characterization of viromes.

Following community statistical analyses based on the normalized coverages of viral populations across samples were conducted and visualized in R version 4.0.2 (81) using the vegan (82) and ggplot2 (83) packages, respectively: Shannon-Wiener indices, Bray-Curtis distance matrix (84) calculations, and subsequent PCoA, PERMANOVA (using the adonis package) (38), CoCA (using the cocorresp package [40]), and BioENV analysis (39). Previously reported (29) environmental and geochemical variables (Table S1b) were used as input for BioENV. CoCA was performed on microbial and viral relative abundance profiles using the cocorresp package in both symmetric and predictive modes. Significance and degree of covariance in symmetric mode were computed, and the ordination biplots were visualized using a method described previously by Alric et al. (85). All permutation-based tests were conducted with 999 iterations. Microbial relative abundance profiles were calculated using read-mapping-based abundances of *rpS3* genes from our previous study (29).

### Intergenomic distance clustering and phylogenetic analysis of putative viruses.

Intergenomic distances of viruses were calculated to identify genus- and species-level clusters using VIRIDIC with default settings (41). Phylogenetic trees were constructed using nucleic acid sequence-based VICTOR (42). vConTACT2 v0.9.19 (43, 86) was used to cluster and classify selected viral scaffolds against the ProkaryoticViralRefseq v94 database (87); resulting clusters were subsequently visualized using Cytoscape v3.8.0 (88). Per-sample relative abundances of viral clusters were calculated by summing up the calculated coverages for each viral population genome in the cluster and then dividing the total by the total coverage of all viral population genomes in the respective sample.

### CRISPR-Cas analysis and spacer extraction of medium- to high-quality host genomes.

For each high-quality host genome, direct repeats and Cas genes associated with CRISPR systems were extracted by combining the tools PILER-CR v1.06 (89) in default settings and CRISPRCasFinder (90), with results filtered for evidence level 4 for the latter. Filtered direct repeats were subsequently used for spacer extraction using MetaCRAST (91) with the flags -d 3 -l 60 -c 0.9 -a 0.9 -r from the raw reads of the respective metagenome from which the MAG was binned. Spacers were dereplicated using CD-HIT v4.6 (77) at 100% identity to identify the number of unique spacers across all metagenomes and within each sample.

### Host-virus matching. (i) Protospacer-to-spacer matching.

Extracted CRISPR spacers from all metagenomes were subjected to BLAST searching (92) with the blastn –short algorithm against the predicted viral sequences across all metagenomes and filtered with an 80% similarity threshold (similarity = alignment length × identity/query length).

### (ii) Oligonucleotide frequency-based matching.

VirHostMatcher (44) was used to determine putative interactions between medium- to high-quality MAGs from all metagenomes and predicted viral genomes based on shared oligonucleotide frequency pattern (*k* = 6). A d_2_* dissimilarity threshold of <0.2 was used to filter all potential host-virus interactions based on the benchmarking performed by Ahlgren et al. (44), where the lowest dissimilarity score threshold of 0.2 yielded above 90% accuracy in host prediction at the class level and approximately 60% accuracy at the order level.

### (iii) tRNA identity-based matching.

tRNAs in viral genomes and MAGs were predicted using DRAM-v (47) and tRNAscan-SE (93), respectively. Viral tRNAs were subjected to BLAST searching (92) against microbial tRNAs, and only complete (100% identity) matches were considered to imply host-virus interactions.

### (iv) Nucleotide sequence homology-based matching.

Viral genomes were subjected to BLAST searching against medium- to high-quality MAGs with a cutoff of ≥75% coverage over the length of the viral contig, ≥70% minimum nucleotide identity, ≥50 -bit score, and E value of ≤0.001. Exact matches resulting from a viral genome being binned in a MAG were excluded as potential binning errors, except when the viral genome was identified by VirSorter to be a defined prophage region inside a longer scaffold. Identified interactions were combined, and the host-virus interaction network was visualized using Cytoscape v3.8.0 (88).

### Genomic neighborhood visualization and phylogenetic analyses of viral genes.

Genomic neighborhoods were compared and visualized using Geneious v11.1.5. software (https://www.geneious.com) and Easyfig v2.2.2 (94) with a BLASTn E-value threshold of 1E−5. Three viral spherulation-specific proteins and 12 viral WhiB-like protein amino acid sequences were queried against the NCBI nr database using BLASTP. Twenty highest-identity matches per spherulation-specific sequence and five highest-identity matches per WhiB-like sequence were selected for subsequent phylogenetic analyses. Spherulation-specific proteins were searched for in the medium- to high-quality MAGs using hmmsearch (HMMER v3.2; www.hmmer.org) with spherulin4.hmm with an E-value threshold of 1E−10. Bacterial WhiB-like protein sequences were selected from the medium- to high-quality MAGs based on their annotation against the UniRef100 database (75). Duplicate sequences were removed prior to alignment using MUSCLE v3.8.31 (95). Spherulation protein alignments were trimmed using BMGE v1.12 (96) due to the presence of larger gaps. Trees were constructed using iqtree v1.5.5 (97) with the flags -m MFP -alrt 1000 -bb 1000 and visualized using iToL (98). Branches with bootstrap (99) values at least 95 and SH-aLRT (100) test values of at least 80 were marked as strongly supported. Phage promoters were predicted using PromoterHunter from phiSITE (101). Rho-dependent and Rho-independent terminators were predicted using RhoTermPredict (102) and ARNold (103), respectively.

### Data availability.

MAGs and viral genomes used in the analyses have been deposited in NCBI under BioProject no. PRJNA665391. NCBI accession information for the viral genomes and MAGs are found in Table S1a and e, respectively.
