# Supplementary figures and images for: Diverse Viruses Carrying Genes for Microbial Extremotolerance in the Atacama Desert Hyperarid Soil
Source: mSystems. 2021 May 18;6(3):e00385-21. doi: 10.1128/mSystems.00385-21 (PMC8269230; doi:10.1128/mSystems.00385-21)

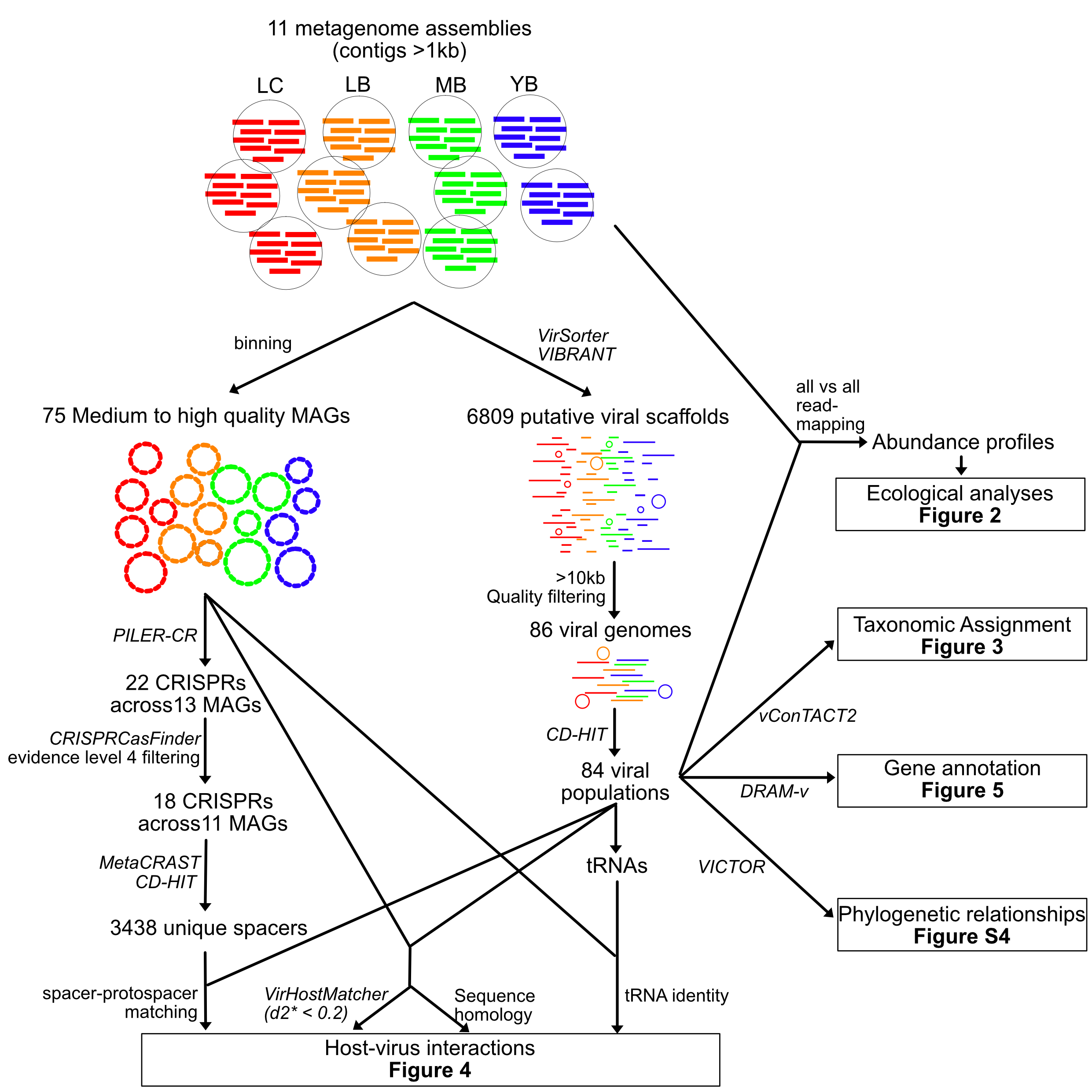

Supplement: FIG S1 [file msystems.00385-21-sf001.tif]

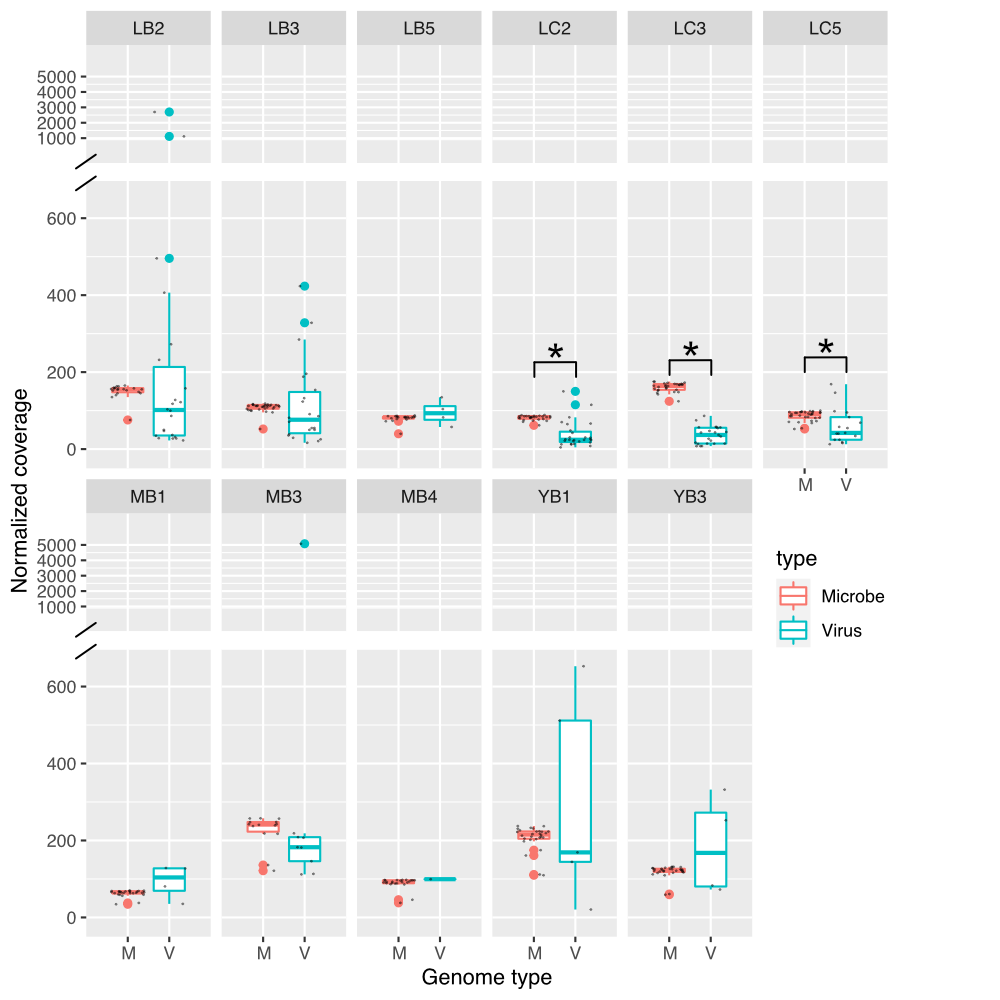

Supplement: FIG S2 [file msystems.00385-21-sf002.tif]

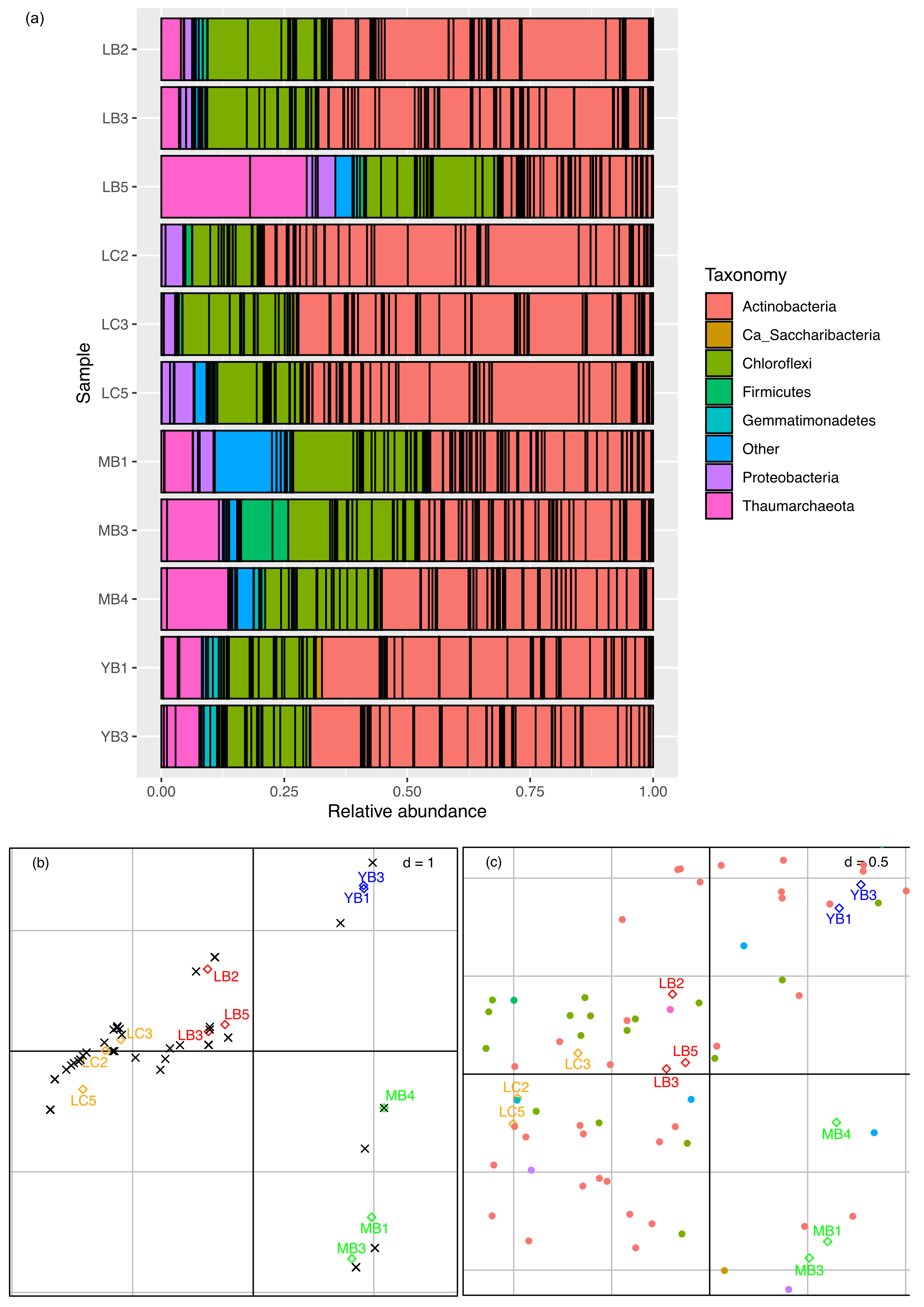

Supplement: FIG S3 [file msystems.00385-21-sf003.tif]

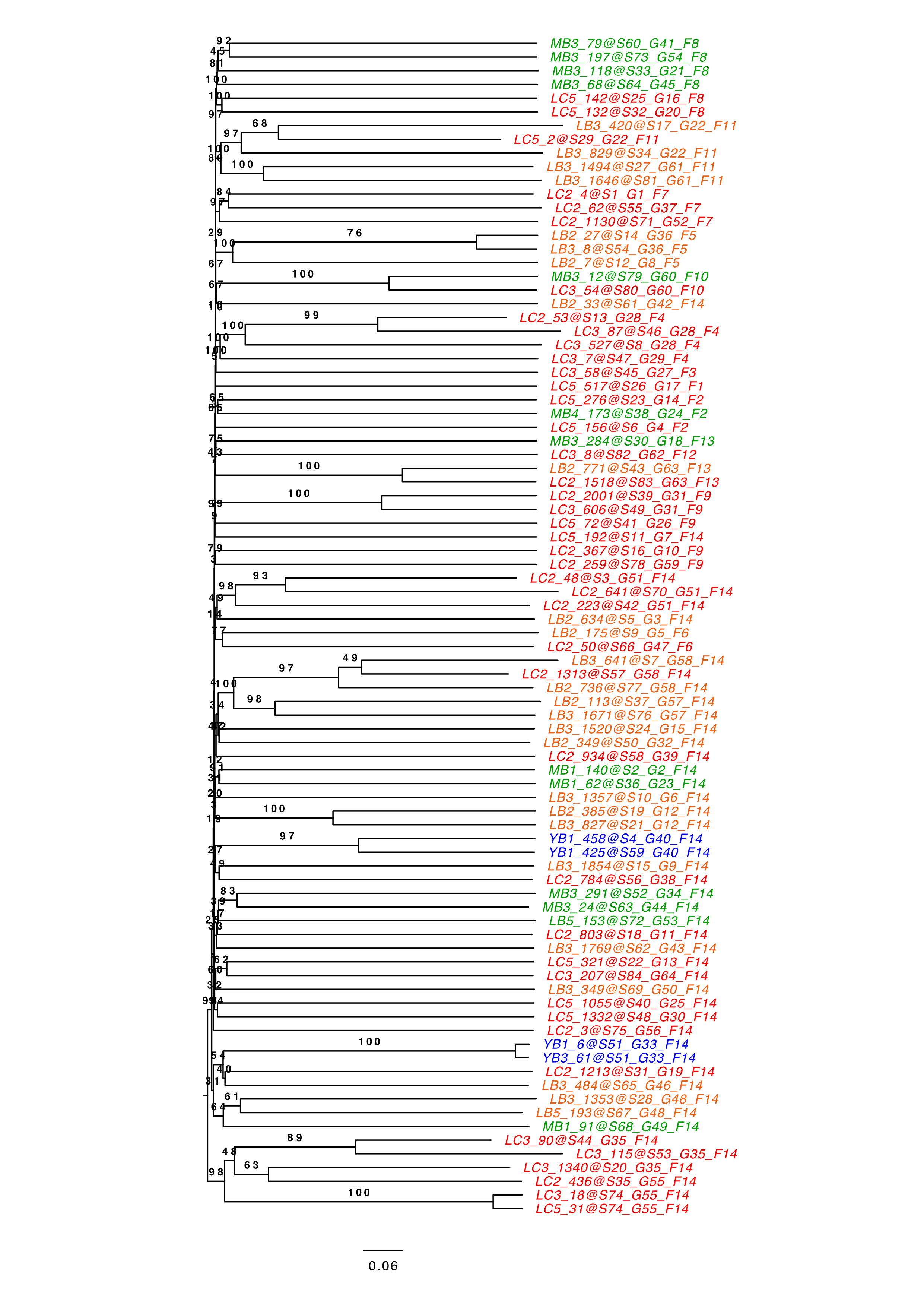

Supplement: FIG S4 [file msystems.00385-21-sf004.tif]

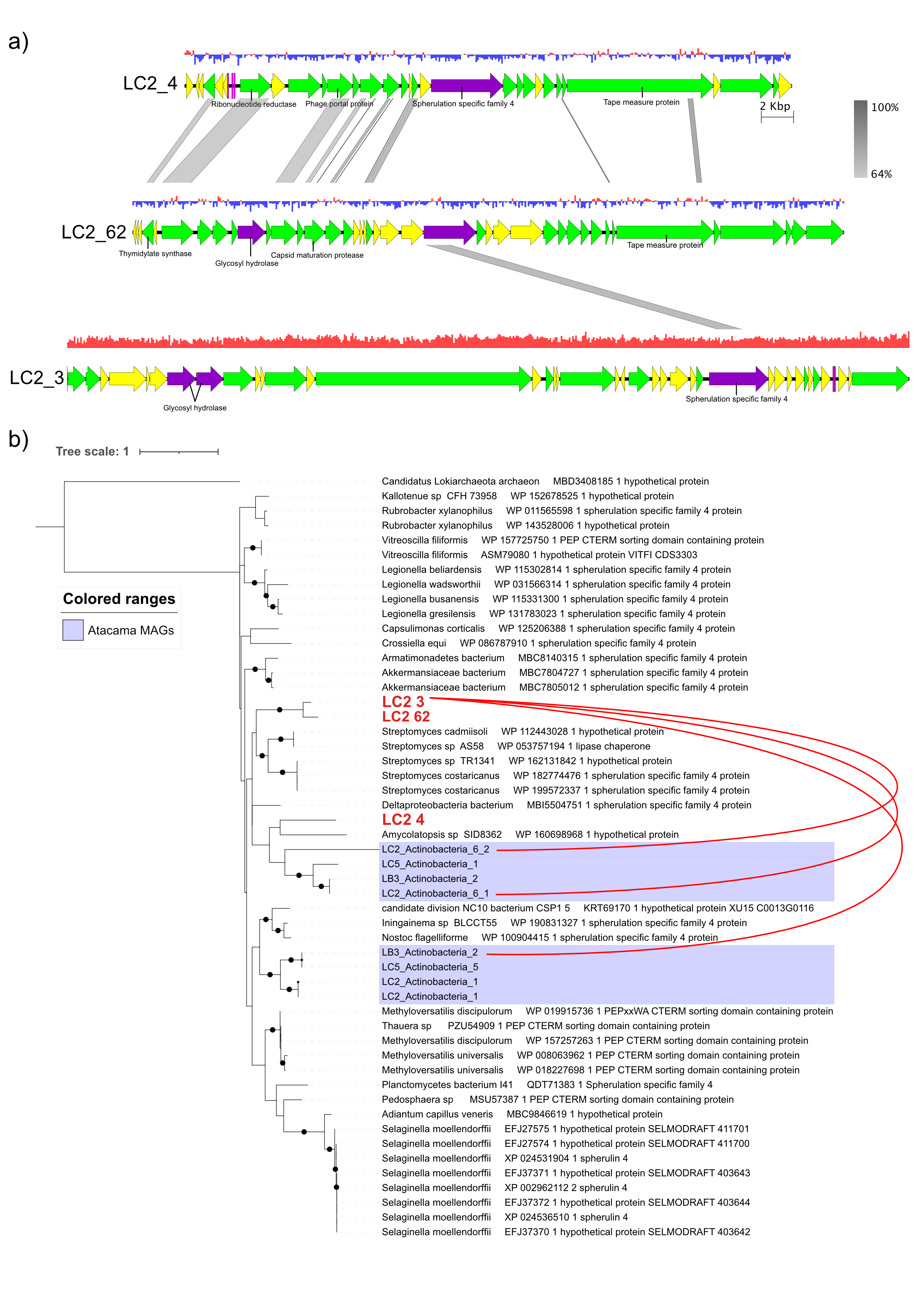

Supplement: FIG S5 [file msystems.00385-21-sf005.tif]

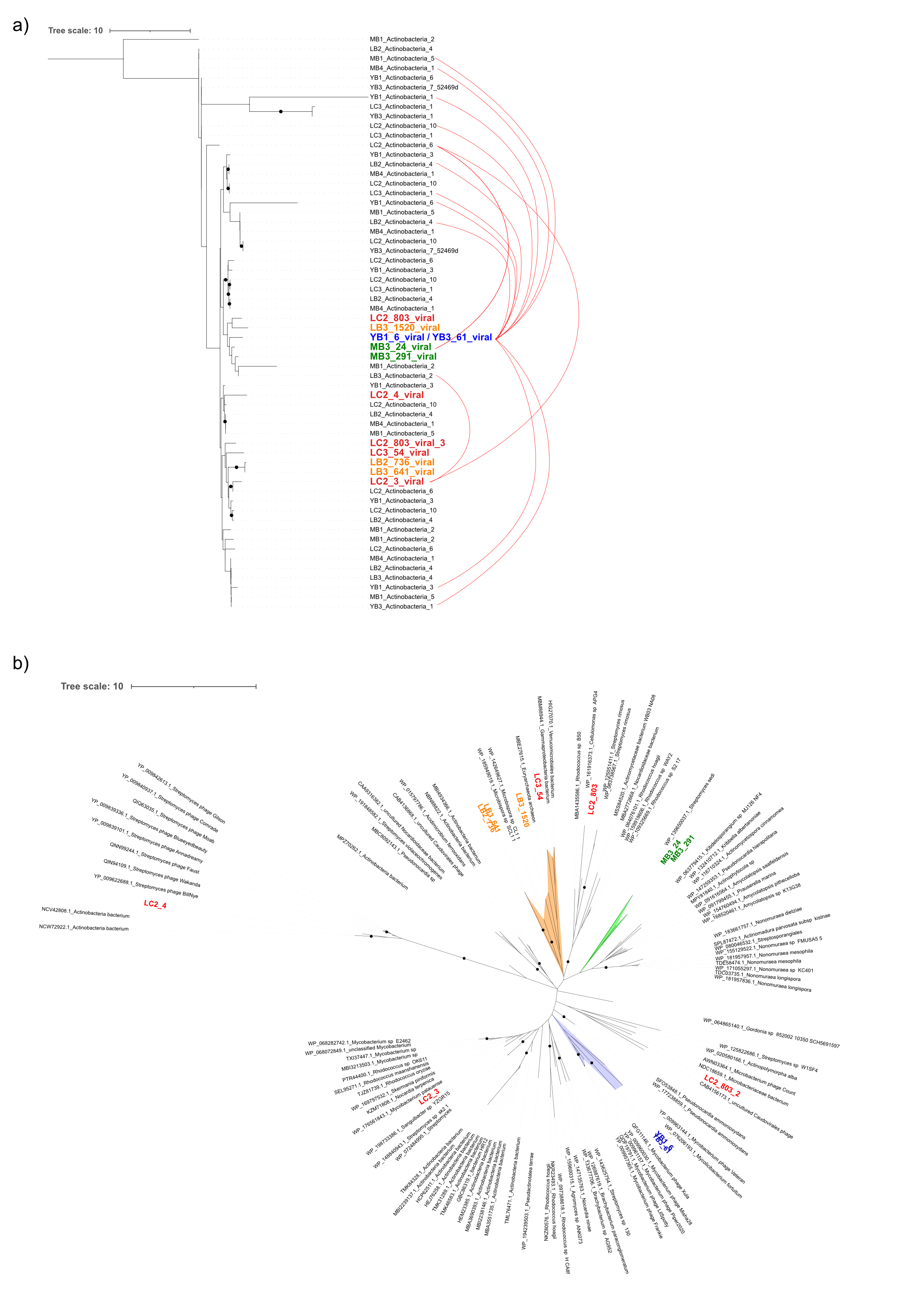

Supplement: FIG S6 [file msystems.00385-21-sf006.tif]
