# Supplementary material for: Diverse Viruses Carrying Genes for Microbial Extremotolerance in the Atacama Desert Hyperarid Soil
Source: mSystems. 2021 May 18;6(3):e00385-21. doi: 10.1128/mSystems.00385-21 (PMC8269230; doi:10.1128/mSystems.00385-21)
Supplement: TEXT S1 [file msystems.00385-21-s001.docx]

**Supplementary Results and Discussion**

***Preliminary analysis of innate antiphage defense systems in MAGs.*** Many of the analyzed MAGs did not have CRISPR-Cas adaptive defense systems. To understand the non-CRISPR-Cas defense mechanisms these extremotolerant microbes might use to protect themselves against diverse and abundant viruses, we examined MAGs for genes involved in innate antiphage defense systems. Of the nine (Restriction-Modification (RM), DISARM, BREX, Bruantia, Abortive Infection (Abi), Zorya, Septu, Gabija, Theoris) innate antiphage defense systems [(1–5)](https://paperpile.com/c/SxFYFJ/7rJhF+Ez1Yn+zpLss+yuDw0+zLepE) surveyed we found RM and Abi to be the most widespread mechanism across the 73 MAGs (**Table S1f**). We found 264 genes involved in the RM system across 53 MAGs belonging to *Actinobacteria*, *Chloroflexi*, *Firmicutes* and *Thaumarchaeota*. Additionally, we found 158 genes involved in the Abi system across 50 MAGs. In total, 64 MAGs contained at least one gene associated with either RM or Abi system with 39 MAGs putatively possessing both systems. Although we identified 12 genes carrying a *pglZ*-domain (one of the genes in the BREX locus [(3)](https://paperpile.com/c/SxFYFJ/zpLss)) in 10 MAGs, no other key BREX or DISARM genes could be identified. This is in contrast to a recent cold analog study [(1)](https://paperpile.com/c/SxFYFJ/7rJhF) that reported a much more diverse set of innate immune systems. However, *pglZ-domains* have been found to be enriched in genomic regions called “defense islands” [(6)](https://paperpile.com/c/SxFYFJ/rup00) harboring phage resistance genes, many of which are yet to be characterized. Thus, these 10 MAGs with *pgIZ*-domain containing genes may possess currently unknown defense mechanisms. Interestingly, MAGs with CRISPR arrays contained more innate anti-phage genes compared to those without any binned CRISPRs (Welch's t-test; p = 0.00946, n = 73). This uneven distribution of both innate and adaptive antiphage systems, with microbes with CRISPRs also carrying more innate antiphage genes than those without CRISPRs, suggests that the viral predation stress varies for each host and elicits different immune responses. Interestingly, we also found a gene of the *HicB* family in a viral genome LB3_1520. *HicB* codes for the antitoxin part of the Type II toxin-antitoxin Abi antiphage system. Phages have been shown to counter the host Abisystem by encoding a mimicked antitoxin gene [(7)](https://paperpile.com/c/SxFYFJ/5Ms95) and this may be evidence of some Atacama viruses adapting to host defense systems.

**References**

1. [Bezuidt OKI, Lebre PH, Pierneef R, León-Sobrino C, Adriaenssens EM, Cowan DA, Van de Peer Y, Makhalanyane TP. 2020. Phages Actively Challenge Niche Communities in Antarctic Soils. mSystems 5.](http://paperpile.com/b/SxFYFJ/7rJhF)

2. [Doron S, Melamed S, Ofir G, Leavitt A, Lopatina A, Keren M, Amitai G, Sorek R. 2018. Systematic discovery of antiphage defense systems in the microbial pangenome. Science.](http://paperpile.com/b/SxFYFJ/Ez1Yn)

3. [Goldfarb T, Sberro H, Weinstock E, Cohen O, Doron S, Charpak-Amikam Y, Afik S, Ofir G, Sorek R. 2015. BREX is a novel phage resistance system widespread in microbial genomes. EMBO J 34:169–183.](http://paperpile.com/b/SxFYFJ/zpLss)

4. [Ofir G, Melamed S, Sberro H, Mukamel Z, Silverman S, Yaakov G, Doron S, Sorek R. 2018. DISARM is a widespread bacterial defence system with broad anti-phage activities. Nat Microbiol 3:90–98.](http://paperpile.com/b/SxFYFJ/yuDw0)

5. [Labrie SJ, Samson JE, Moineau S. 2010. Bacteriophage resistance mechanisms. Nat Rev Microbiol 8:317–327.](http://paperpile.com/b/SxFYFJ/zLepE)

6. [Makarova KS, Wolf YI, Snir S, Koonin EV. 2011. Defense islands in bacterial and archaeal genomes and prediction of novel defense systems. J Bacteriol 193:6039–6056.](http://paperpile.com/b/SxFYFJ/rup00)

7. [Blower TR, Short FL, Fineran PC, Salmond GPC. 2012. Viral molecular mimicry circumvents abortive infection and suppresses bacterial suicide to make hosts permissive for replication. Bacteriophage 2:234–238.](http://paperpile.com/b/SxFYFJ/5Ms95)
